# Knowledge, Attitudes, and Practices Regarding Breast Cancer and Its Prevention Among Female Doctors at a Tertiary Care Hospital in Pakistan

**DOI:** 10.7759/cureus.100962

**Published:** 2026-01-06

**Authors:** Safina Tanveer, Faiza Gul, Asma Rasool Peerzada, Ayesha Johar, Nida Mumtaz, Naveedul Haq, Safwa Nayab, Muhammad Bilal Elahi, Nawal Qadus, Ikram Ullah

**Affiliations:** 1 General Surgery, Khyber Teaching Hospital, Peshawar, PAK; 2 General Surgery, Khyber Medical College, Peshawar, PAK; 3 Internal Medicine, Khalifa Gul Nawaz Teaching Hospital, Peshawar, PAK; 4 Internal Medicine, Khyber Teaching Hospital, Peshawar, PAK; 5 Internal Medicine, Khyber Medical College, Peshawar, PAK; 6 Internal Medicine, Lady Reading Hospital, Peshawar, PAK; 7 General Surgery, MMC (Maqsood Medical Complex) General Hospital, Peshawar, PAK; 8 Internal Medicine, Northampton General Hospital, Northampton, GBR; 9 Acute Medicine, Northampton General Hospital, Northampton, GBR

**Keywords:** early detection of cancer, general surgery and breast cancer, healthcare provider, knowledge assessment, mammography

## Abstract

Background and objectives

Breast cancer is the leading cause of cancer mortality among women, yet screening remains underutilized in low-resource settings. Despite the central role of physicians in patient counselling, early detection, and guideline implementation, most research in Pakistan about breast cancer awareness has focused on nurses, medical students, or the general population. Evidence specifically examining female doctors, particularly in Khyber Pakhtunkhwa province, remains sparse. This study assessed breast cancer knowledge, attitudes, and screening practices among female doctors at a tertiary hospital in Peshawar, examined associations between knowledge and screening behavior, and identified perceived barriers to screening.

Methods

A cross-sectional survey was conducted among 188 female doctors at Khyber Teaching Hospital, Peshawar, Pakistan, in October 2025. Data were collected using a structured, self-administered questionnaire. The instrument assessed knowledge across three domains (risk factors, signs and symptoms, and screening methods) comprising 27 items, attitudes toward breast cancer screening using a six-item Likert scale, current screening practices, and perceived barriers to screening. Statistical analyses included Spearman's correlation to examine relationships between knowledge, attitudes, and practices; Chi-square tests to assess associations between categorical variables; and Mann-Whitney U tests to compare knowledge scores across demographic groups.

Results

Participants had a mean age of 27.47 ± 1.53 years. Knowledge varied substantially across domains: 94.1% demonstrated good knowledge of signs and symptoms, compared to 9.6% for risk factors and 2.1% for screening methods. Attitudes were overwhelmingly positive (mean score: 29.01 ± 1.72 out of 30). Despite favorable attitudes, screening practices were suboptimal: 63.8% practiced breast self-examination (BSE), although only 36.7% performed it monthly; 26.6% had undergone clinical breast examination; and 2.7% reported mammography. Knowledge scores did not differ significantly between BSE practitioners and non-practitioners (p=0.409). Family history of breast cancer was significantly associated with BSE practice (p=0.013), as was lack of guideline awareness with non-practice (p=0.010). The most frequently reported barriers were busy schedules (71.3%), perceived low risk (41.5%), and absence of institutional screening programs (32.4%).

Conclusion

Female doctors demonstrated strong symptom recognition and positive attitudes but had limited knowledge of screening guidelines and suboptimal screening practices. The lack of association between knowledge and practice suggests that educational interventions alone are insufficient. Strategies to improve screening adherence among female doctors should include workplace-based screening programs, continuing medical education on breast health, and targeted counselling for those with identifiable risk factors.

## Introduction

In 2020, breast cancer surpassed lung cancer to become the most commonly diagnosed malignancy worldwide and the leading cause of cancer mortality among women [1]. Although age-standardized incidence rates of breast cancer remain lower in Asia and Africa compared to Western nations, these regions account for nearly 72% of global breast cancer deaths [2]. This disparity reflects limited screening infrastructure, delayed diagnosis, and constrained treatment resources, resulting in substantially higher mortality-to-incidence ratios and late-stage presentation at diagnosis [3].

In Pakistan, one in four female cancer deaths is attributable to breast cancer, and between 1990 and 2019, incidence increased by over 300%, while mortality rose by 200%-300% [4]. Karachi, the country's largest metropolitan center, reports the highest breast cancer incidence in Asia [5]. These trends underscore the urgent need for enhanced awareness and early detection strategies.

Early detection significantly improves breast cancer survival. According to the American Cancer Society, established risk factors include increasing age, personal or family history, benign breast disease, dense breast tissue, early menarche, late menopause, hormone replacement therapy, obesity, and delayed or absent childbearing [6]. Common presenting symptoms include nipple discharge or bleeding, nipple inversion, changes in breast or nipple shape, skin dimpling, and any lump or thickening in the breast or axilla. Recognition of these risk factors and warning signs is fundamental to detecting disease at a treatable stage [7].

Three established screening modalities exist for early detection: breast self-examination (BSE), clinical breast examination (CBE), and mammography [8]. Mammography remains the gold standard, although its sensitivity is reduced in younger women due to breast tissue density [9]. The American Cancer Society recommends initiating screening at age 40, with annual mammography from age 45 [10]. However, disease patterns in Pakistan differ from those in the West; younger women are increasingly diagnosed despite having no identifiable risk factors [11]. This necessitates earlier screening, with annual mammography recommended from age 40 [11].

Despite the availability of effective screening methods, most Pakistani breast cancer patients present at advanced stages, primarily due to a lack of awareness and low socioeconomic status [12]. Pakistan allocates approximately 2.9% of its GDP to health, well below the global average of 7.2% [13,14], and screening facilities remain scarce: only 9.5% of urban and 4.8% of rural women utilize screening services, while radiological facilities are accessible to just 2.5% and 0.7%, respectively [8]. Where infrastructure for mammography is limited, awareness of risk factors, symptoms, and self-examination becomes critical. Given these constraints, improving breast cancer awareness represents the most feasible and cost-effective strategy for promoting early detection. However, beyond knowledge deficits, social and cultural barriers that delay help-seeking must also be addressed [12]. Identifying these gaps requires assessing Knowledge, Attitudes, and Practices (KAP), a framework endorsed by the World Health Organization for evaluating health awareness and behavior at the population level [15].

Despite the central role of physicians in patient counselling, early detection, and guideline implementation, studies assessing breast cancer knowledge and screening behavior in Pakistan have largely focused on nurses, medical students, or the general population. Evidence examining female doctors, particularly in Khyber Pakhtunkhwa province, remains sparse. This study aimed to (1) assess breast cancer knowledge across domains of risk factors, signs and symptoms, and screening methods; (2) evaluate attitudes toward breast cancer prevention and screening; (3) determine the prevalence and patterns of screening practices including BSE, CBE, and mammography; (4) examine the relationship between knowledge, attitudes, and screening behavior; and (5) identify perceived barriers to screening among female doctors at a tertiary care hospital in Peshawar, Pakistan.

## Materials and methods

A descriptive cross-sectional survey was conducted in October 2025 among female doctors to evaluate their understanding of breast cancer. The study received approval from the Institutional Research and Ethical Review Board of Khyber Medical College, Peshawar, Pakistan (Approval No. 936/DME/KMC, dated: 06-10-2025). The target population consisted of registered female doctors working in various departments of Khyber Teaching Hospital, Peshawar. Sample size was calculated using the OpenEpi (Centers for Disease Control and Prevention (CDC) and Rollins School of Public Health, Emory University, Atlanta, Georgia, USA) sample size calculator for proportions, assuming a 95% confidence level, 5% margin of error, and a conservative estimate of 50% prevalence of adequate knowledge to maximize sample size. After inflating by 10% to account for non-response, the final required sample size was 223 participants.

Inclusion criteria comprised female doctors aged 25-55 years, working in the hospital, and willing to participate. Exclusion criteria included female paramedical staff, incomplete responses, and those who did not provide consent. A total of 188 completed responses were received within the designated data collection period, yielding a response rate of 84.3%. The remaining participants did not return the questionnaire within the stipulated timeframe and were excluded from analysis. Participants were recruited through non-probability convenience sampling. Female doctors were contacted via institutional email and WhatsApp (WhatsApp Messenger, Meta Platforms, Inc. (formerly Facebook, Inc.), Menlo Park, California, USA) and informed about the study objectives. Participation was voluntary, and electronic informed consent was obtained before commencing the survey.

The authors developed a structured, self-administered questionnaire after reviewing established literature on breast cancer risk factors, symptoms, screening recommendations, preventive behaviors, and barriers to screening. The questionnaire was pretested among 10 female doctors for clarity and comprehension, and minor modifications were made based on their feedback before data collection (Appendix A). A brief introduction and consent statement were provided at the beginning of the questionnaire, ensuring participants' confidentiality and voluntary participation. The instrument was divided into five sections: (1) demographic information, (2) knowledge assessment, (3) attitudes toward breast cancer prevention, (4) screening practices, and (5) perceived barriers to screening.

Section two assessed knowledge through 27 items across three domains: risk factors (10 items), signs and symptoms (five items), and screening methods (12 items). Each correct response scored 1 point; incorrect or don't know responses scored 0. A total score of <50% was categorized as poor knowledge, 50%-75% as fair knowledge, and >75% as good knowledge. Correct responses for knowledge items were determined based on established guidelines appropriate for the study context. Risk factor items were scored according to the American Cancer Society classification of breast cancer risk factors [6], which categorizes potential risk factors into four tiers: non-modifiable established factors, lifestyle-related established factors, factors with unclear effects, and disproven factors. Only factors classified within the first two tiers were scored as 'Yes' and regarded as established risk factors. For example, 'Cigarette smoking increases the risk of breast cancer' was scored as 'No' because the American Cancer Society (ACS) lists smoking under 'Factors with Unclear Effects.' Signs and symptoms items were based on the National Breast Cancer Foundation (NBCF) [7]. Screening method items were scored according to Pakistan's National Action Plan for Prevention and Control of Non-Communicable Diseases and Health Promotion (NAP-NCD) [8], which recommends breast self-examination, clinical breast examination, and mammography as appropriate screening modalities for early detection of breast cancer in Pakistan.

Section three assessed attitudes toward breast cancer prevention using six items rated on a five-point Likert scale (1=strongly agree to 5=strongly disagree). Responses were reverse-coded so that higher scores indicate more positive attitudes and summed to yield a total attitude score (range: 6-30). Section four assessed screening practices, including BSE (yes/no; if yes: frequency), CBE (yes/no; if yes: routine vs. symptomatic), mammography (yes/no), body weight monitoring, and physical activity levels. Section five assessed perceived barriers through a predefined checklist of 11 items. Respondents could select all applicable barriers. Each item was coded as a binary variable for analysis.

Statistical analysis

All responses were automatically compiled via Google Forms (Google LLC, Mountain View, California, USA) and analyzed using IBM SPSS Statistics version 31 (IBM, Armonk, New York, USA). Descriptive statistics, including frequencies and percentages, were used to summarize categorical variables, while means and standard deviations were calculated for continuous variables. Spearman's rank correlation coefficients were computed to examine relationships between knowledge, attitudes, and practices. Chi-square tests were performed to assess associations between categorical variables. Mann-Whitney U tests were used to compare knowledge and attitude scores between groups. All tests were two-tailed, and p<0.05 was considered statistically significant.

## Results

A total of 188 female doctors participated in the study, with a mean age of 27.47 ± 1.53 years (range: 24-33 years). The majority were postgraduate residents (n=157; 83.5%), while 31 (16.5%) were house officers. Participants represented a broad range of specialties. Out of 188, 119 (63.3%) participants were unmarried, 67 (35.6%) were married, and one participant each (0.5%) was divorced and widowed. Regarding family history, 49 participants (26.1%) reported a family history of breast cancer: 17 (9.0%) in a first-degree relative and 37 (19.7%) in a second-degree relative, with five reporting both. The majority (n=139; 73.9%) had no family history. Table 1 presents the detailed demographic characteristics.

**Table 1:** Demographic Characteristics of Study Population (N=188) *Among participants with positive family history (n = 49): first-degree relatives, 17 (34.7%), and second-degree relative, 37 (75.5%). Categories for the degree of affected relatives are not mutually exclusive. GP: general practitioner, OBGYN: obstetrics and gynecology.

Characteristic	Category	N (%)
Age (years)	Mean ± SD (Range)	27.47 ± 1.53 (24-33)
Designation	House officer	31 (16.5%)
	Postgraduate resident	157 (83.5%)
Department	Surgery	66 (35.1%)
	Medicine	44 (23.4%)
	OBGYN	31 (16.5%)
	Pediatrics	14 (7.4%)
	Ophthalmology	10 (5.3%)
	Radiology	10 (5.3%)
	Dermatology	6 (3.2%)
	Psychiatry	4 (2.1%)
	Anesthesiology	3 (1.6%)
Marital status	Unmarried	119 (63.3%)
	Married	67 (35.6%)
	Divorced/Widowed	2 (1.1%)
Family history	Yes	49 (26.1%)
	No	139 (73.9%)
	First-degree relative*	17 (9.0%)
	Second-degree relative*	37 (19.7%)
Registered with GP	Yes	48 (25.5%)
	No	135 (71.8%)
	Prefer not to say	5 (2.7%)

Knowledge assessment

Knowledge levels varied across the three assessed domains. The mean total knowledge score was 15.90 ± 2.73 out of a maximum of 27 points (58.9%). Knowledge of signs and symptoms was high, with a mean score of 4.76 ± 0.63 out of 5, and 177 out of 188 (94.1%) participants demonstrated good knowledge in this domain. Knowledge of screening methods was lower, with a mean score of 5.47 ± 1.92 out of 12; only four of 188 (2.1%) achieved good knowledge, while 99 of 188 (52.7%) demonstrated poor knowledge. Table 2 presents the knowledge score distributions by domain, and Table 3 presents the item-wise knowledge assessment.

**Table 2:** Knowledge Score Distributions by Domain (N=188)

Knowledge Domain	Mean ± SD	Good N (%)	Fair N (%)	Poor N (%)
Risk factors (max: 10)	5.67 ± 1.45	18 (9.6%)	82 (43.6%)	88 (46.8%)
Signs and symptoms (max: 5)	4.76 ± 0.63	177 (94.1%)	8 (4.3%)	3 (1.6%)
Screening methods (max: 12)	5.47 ± 1.92	4 (2.1%)	85 (45.2%)	99 (52.7%)
Total knowledge (max: 27)	15.90 ± 2.73	6 (3.2%)	154 (81.9%)	28 (14.9%)

**Table 3:** Item-Wise Knowledge Assessment (N=188) *Correct answer refers to responses aligned with established guidelines. Risk factor items scored per ACS classification [6]; factors with unclear evidence (e.g., cigarette smoking) scored as 'No.' Signs/symptoms based on NBCF [7]. Screening methods scored per Pakistan's NAP-NCD [8]. NBCF: National Breast Cancer Foundation, NAP-NCD: National Action Plan for Prevention and Control of Non-Communicable Diseases and Health Promotion, ACS: American Cancer Society.

Knowledge Domain	Question	Correct Answer *	N (%)
Risk factors	Does having a history of breast cancer increase the risk of developing it again?	Yes	183 (97.3%)
	Does using Hormone Replacement Therapy increase the risk of breast cancer?	Yes	148 (78.7%)
	Cigarette smoking increases the risk of breast cancer.	No	164 (87.2%)
	Being overweight (BMI over 25) increases the risk of breast cancer?	Yes	118 (62.8%)
	Does having dense breast tissue on a mammogram increase the risk of breast cancer?	Yes	123 (65.4%)
	Having children later on in life or not at all increases the risk?	Yes	135 (71.8%)
	Increasing age is a risk factor for breast cancer.	Yes	149 (79.3%)
	Use of antiperspirants increases the risk of breast cancer.	No	120 (63.8%)
	Disruptions in circadian rhythm (night shift work) increase the risk of breast cancer.	No	112 (59.6%)
	<30 mins of moderate physical activity, 5 times a week, increases the risk of breast cancer.	Yes	64 (34.0%)
Signs and Symptoms	Nipple Discharge	Yes	174 (92.6%)
	Breast Skin Dimpling	Yes	182 (96.8%)
	Pulling in of Nipple	Yes	177 (94.1%)
	Change in the breast or nipple size and shape	Yes	184 (97.9%)
	Lump in the breast or armpit	Yes	184 (97.9%)
Screening Methods	How many recognized screening methods are there for early detection of breast cancer?	3	124 (66.0%)
	The recognized screening methods?	BSE, CBE, Mammography	18 (9.5%)
	At what age should women begin BSE?	20 years	107 (56.9%)
	How often should women perform BSE?	Monthly	119 (63.3%)
	What is the best time to perform BSE?	1 week after menstrual period	88 (46.8%)
	At what age should women begin CBE?	20 years	86 (45.7%)
	How often should women perform CBE?	Yearly	86 (45.7%)
	At what age should women with average risk begin mammography screening?	40 years	79 (42.0%)
	How often should mammography be performed by women?	Yearly	112 (59.6%)

Attitudes toward breast cancer prevention

**Figure 1:**
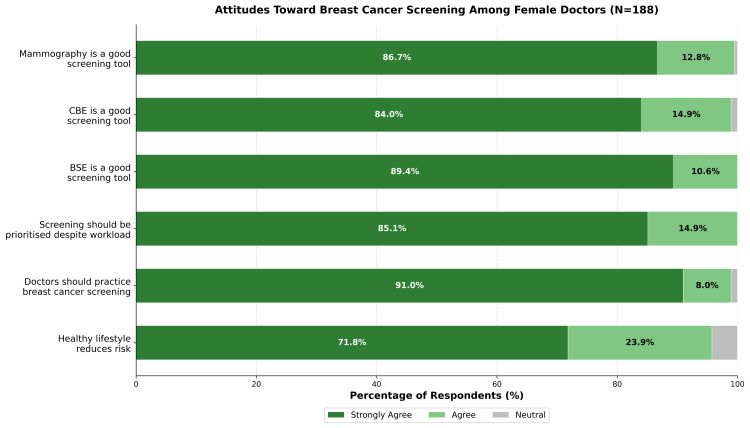
Distribution of Attitude Responses Toward Breast Cancer Prevention (N=188) Horizontal stacked bar chart showing distribution of responses on a five-point Likert scale. No respondents selected 'Disagree' or 'Strongly Disagree' for any attitude item. CBE: clinical breast examination, BSE: breast self-examination.

Participants demonstrated positive attitudes toward breast cancer prevention, with a mean total score of 29.01 ± 1.72 out of 30 (range: 23-30). A ceiling effect was observed, with 112 of 188 (59.6%) achieving the maximum score and 166 of 188 (88.3%) scoring ≥28. Figure 1 illustrates the distribution of responses.

Breast cancer screening practices

**Figure 2:**
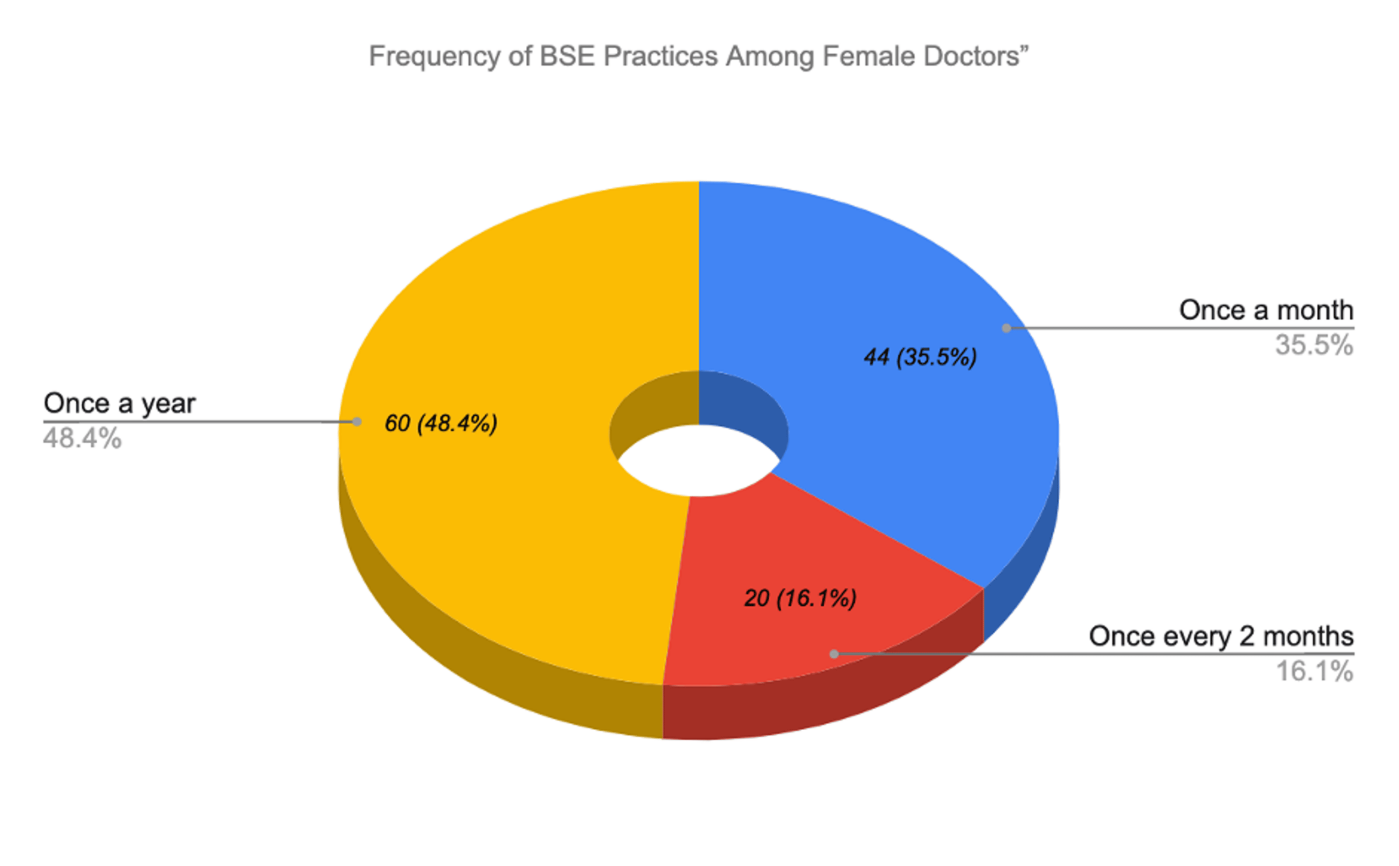
Frequency of BSE Among Female Doctors BSE: breast self-examination.

**Figure 3:**
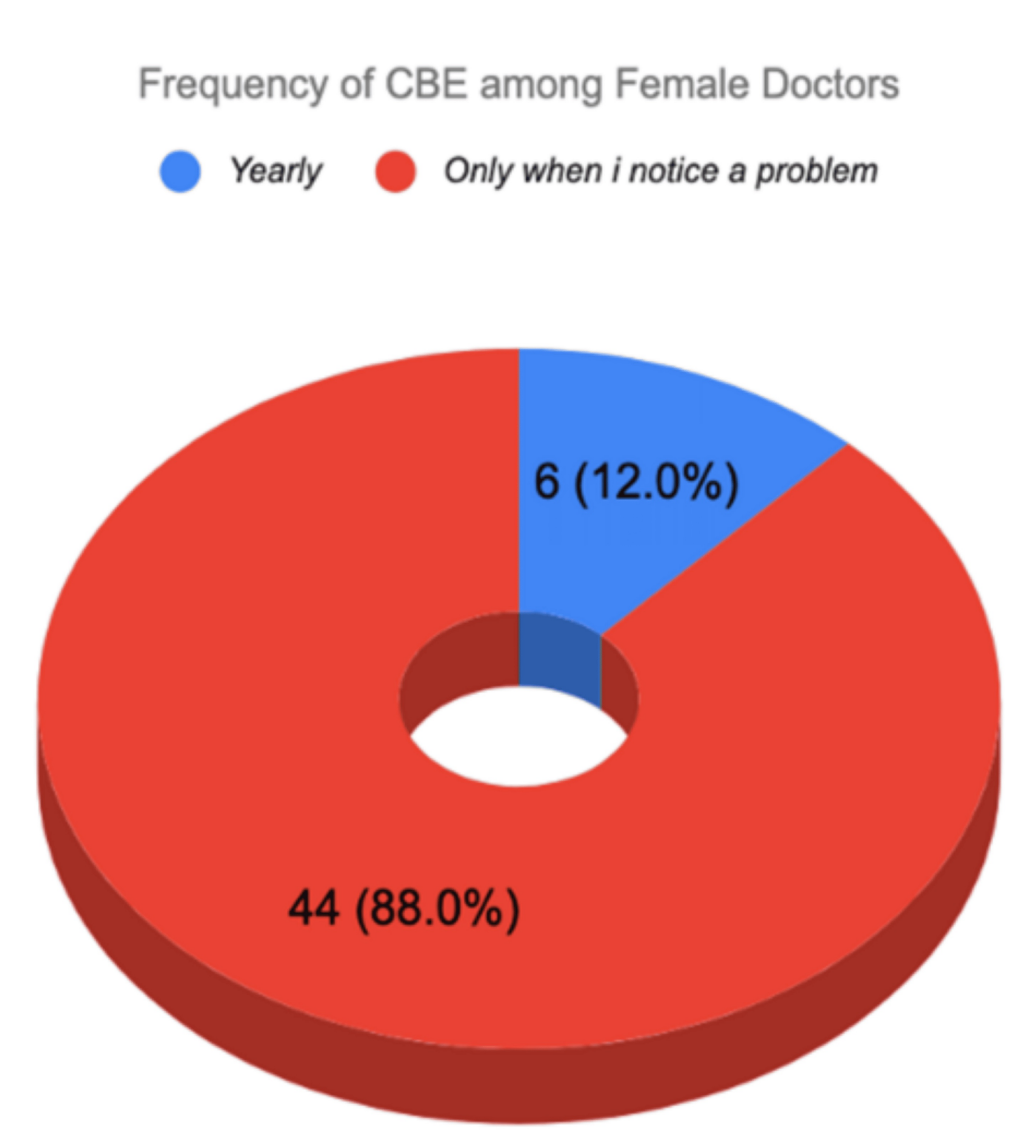
Frequency of CBE Among Female Doctors CBE: clinical breast examination.

BSE was practiced by 120 of 188 (63.8%) participants. Among those who practiced BSE, frequency varied: 44 of 120 (36.7%) performed it monthly, 20 of 120 (16.7%) every two months, and 56 of 120 (46.7%) yearly or less frequently. CBE uptake was lower, with only 50 of 188 (26.6%) having ever undergone the procedure. Among those who had CBE, 44 of 50 (88.0%) did so only when a problem was noticed rather than as routine screening, while only six of 50 (12.0%) had routine CBE. Mammography was reported by five of 188 (2.7%) participants. No participants were aged ≥40 years, the threshold at which routine mammography screening is recommended for average-risk women. The questionnaire did not distinguish between routine and diagnostic mammography, which represents a study limitation. Regarding general health practices, 150 of 188 (79.8%) reported monitoring their body weight. Regarding physical activity, 75 of 188 (39.9%) engaged in 30 or more minutes of physical activity always or often, while 113 of 188 (60.1%) did so sometimes or never. Table 4 and Figures 2, 3 illustrate BSE practice and frequency distribution.

**Table 4:** Self-reported Practices Among Female Doctors for Breast Cancer Prevention

Practices	Response	N (%)
Do you actively monitor body weight?	Yes	150 (79.8%)
	No	38 (20.2%)
Do you engage in 30 mins of physical activity at least 5 times a week?	Always	24 (12.8%)
	Often	51 (27.1%)
	Sometimes	55 (29.2%)
	Rarely	46 (24.3%)
	Never	12 (6.3%)
If you have children, did you or would you consider breastfeeding to reduce breast cancer risk?	Yes	171 (91%)
	No	17 (9%)
Do you practice BSE?	Yes	120 (63.8%)
	No	68 (36.2%)
Have you ever had CBE?	Yes	50 (26.6%)
	No	138 (73.4%)
Have you ever had mammography?	Yes	5 (2.7%)
	No	164 (87%)
	Not applicable	19 (10.1%)

Perceived barriers to screening

**Figure 4:**
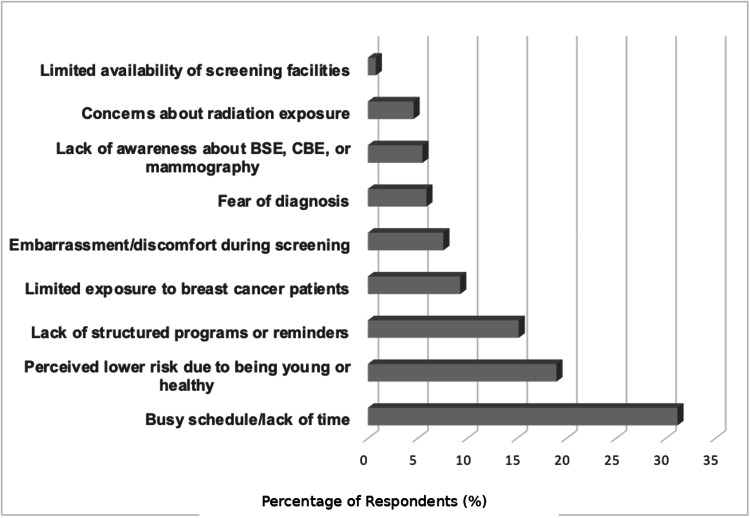
Perceived Barriers to Breast Cancer Screening (N=188) BSE: breast self-examination, CBE: clinical breast examination.

Participants identified multiple barriers to breast cancer screening. The most frequently cited barrier was busy schedule or lack of time (134/188, 71.3%), followed by perceived low risk due to being young or healthy (78/188, 41.5%), absence of structured screening programs or reminders for doctors (61/188, 32.4%), limited exposure to breast cancer cases within their specialty (33/188, 17.6%), embarrassment or discomfort during screening (21/188, 11.2%), fear of receiving a serious diagnosis (20/188, 10.6%), lack of awareness about BSE, CBE, or mammography guidelines (20/188, 10.6%), and concerns about radiation exposure from mammography (13/188, 6.9%). Figure 4 presents the barrier frequencies.

Relationship between knowledge, attitudes, and practices

Spearman’s rank correlation analysis was performed to assess relationships between knowledge domains, attitudes, and BSE frequency. A significant positive correlation was found between signs/symptoms' knowledge and screening knowledge (ρ=0.217, p=0.003). A significant positive correlation was observed between total attitude score and BSE frequency (ρ=0.158, p=0.031). No significant correlation was found between total knowledge score and BSE frequency (ρ=-0.061, p=0.409). Table 5 presents the complete correlation matrix.

**Table 5:** Spearman's Correlation Matrix for Knowledge, Attitudes, and BSE Frequency (N=188) *p < 0.05, **p < 0.01; ρ=Spearman's correlation coefficient. BSE: breast self-examination.

Variables	1	2	3	4	5
1. Risk factor knowledge	—				
2. Signs/symptoms knowledge	0.074	—			
3. Screening knowledge	0.018	0.217**	—		
4. Total attitude score	-0.004	0.016	0.067	—	
5. BSE frequency	-0.001	-0.119	-0.044	0.158*	—

Mann-Whitney U tests revealed no significant differences in knowledge or attitude scores between BSE practitioners (n=120) and non-practitioners (n=68). Total knowledge scores were similar between practitioners (15.95 ± 2.52) and non-practitioners (15.82 ± 3.10; U=4323.5, p=0.494). Similarly, no significant differences emerged for risk factor knowledge (p=0.789), screening knowledge (p=0.257), or total attitude scores (p=0.815). Table 6 presents these comparisons.

**Table 6:** Comparison of Scores by BSE Practice (Mann-Whitney U Test) U=Mann-Whitney U statistic. BSE: breast self-examination.

Variable	BSE Yes (n=120)	BSE No (n=68)	U	p-value
Total knowledge	15.95 ± 2.52	15.82 ± 3.10	4323.5	0.494
Risk factor knowledge	5.67 ± 1.44	5.66 ± 1.49	4174.5	0.789
Screening knowledge	5.56 ± 1.83	5.32 ± 2.07	4481.0	0.257
Total attitude score	29.01 ± 1.70	29.01 ± 1.77	4005.5	0.815

Factors associated with BSE practice

Chi-square analysis was performed to identify factors associated with BSE practice. Family history of breast cancer was significantly associated with BSE practice (χ²=6.238, df=1, p=0.013). Among participants with family history, 79.6% practiced BSE compared to 58.3% without family history. Table 7 presents this contingency analysis.

**Table 7:** Association Between Family History and BSE Practice χ²=6.238, degree of freedom=1, p=0.013. BSE: breast self-examination.

Family History	No BSE n (%)	Yes BSE n (%)	Total
No family history	58 (41.7%)	81 (58.3%)	139
With family history	10 (20.4%)	39 (79.6%)	49

Barrier analysis by BSE practice status

Chi-square analysis was also performed to examine associations between individual barriers and BSE practice. Lack of awareness about screening guidelines was the only barrier significantly associated with non-practice of BSE (χ²=6.720, df=1, p=0.010). Among BSE non-practitioners, 19.1% cited lack of awareness as a barrier compared to only 5.8% of practitioners. Despite being the most frequently cited barrier overall, busy schedule was not significantly associated with BSE practice status (χ²=0.465, p=0.496). Table 8 presents the barrier analysis.

**Table 8:** Association Between Barriers and BSE Practice **p<0.01=statistically significant, χ²: Chi-square value. BSE: breast self-examination, BCa: breast cancer.

Barrier	BSE Yes (%)	BSE No (%)	χ²	p-value
Busy schedule	69.2%	75.0%	0.465	0.496
Perceived low risk	42.5%	39.7%	0.048	0.826
Lack of structured programs	35.0%	27.9%	0.691	0.406
Limited exposure to BCa	16.7%	19.1%	0.051	0.822
Embarrassment	9.2%	14.7%	0.842	0.359
Fear of diagnosis	9.2%	14.7%	0.842	0.359
Lack of awareness**	5.8%	19.1%	6.720	0.010
Radiation concerns	5.0%	11.8%	1.984	0.159

## Discussion

In our study, participants demonstrated strong symptom recognition but poor overall breast cancer knowledge, with only 3.2% achieving good total knowledge scores. This mirrors national findings, where only 35% of female nurses in Karachi possessed adequate breast cancer awareness [16]. It also aligns with international evidence showing that symptom recognition consistently outpaces understanding of risk factors and screening protocols among healthcare workers [17-19]. A systematic review by Meshkani et al. confirmed this trend, noting that even healthcare professionals often demonstrate knowledge gaps comparable to the general population [20]. These findings underscore that medical training alone does not ensure comprehensive breast cancer literacy.

Attitudes toward screening were overwhelmingly positive, with most participants strongly endorsing BSE, CBE, and mammography as essential for early detection. However, a clear disconnect emerged between belief and behavior. While 63.8% reported practicing BSE, the quality of practice was inconsistent: only 36.7% performed it at the recommended monthly frequency, and only 46.8% demonstrated knowledge of correct timing. These findings contrast with Reisi et al., who found that 66.4% of Iranian female health workers performed BSE at the appropriate post-menstrual time [21], suggesting that awareness of both frequency and timing remains a gap in our population.

The low mammography uptake (2.7%) warrants contextualization within current international guidelines. The National Comprehensive Cancer Network (NCCN) recommends that average-risk women begin annual mammography at age 40, with clinical breast examinations every one to three years from age 25 to 39 [22]. Similarly, the European Society for Medical Oncology (ESMO) recommends biennial mammography for women aged 50-69, with conditional recommendations for younger age groups [23]. Given that no participants met the ≥40-year threshold for routine mammography, low uptake is expected for average-risk women. However, 49 participants (26.1%) reported a family history of breast cancer, and guidelines recommend individualized risk assessment for such women, which may include earlier imaging with mammography or MRI [22]. Whether these high-risk individuals received appropriate counseling or screening was beyond the scope of this study. Moreover, the suboptimal CBE uptake is concerning, with only 26.6% of participants having ever undergone the examination. This is particularly notable given that both NCCN and ESMO recommend clinical breast examination for women under 40 as a key component of breast awareness and early detection [22,23].

Total knowledge scores showed no significant association with BSE frequency, and Mann-Whitney U tests revealed no significant difference in knowledge between BSE practitioners and non-practitioners (p=0.494). Similarly, knowledge and attitude scores did not differ between those who practice BSE and those who do not. These findings challenge the conventional assumption that knowledge drives behavior, suggesting that higher knowledge levels do not translate into more frequent screening. Heena et al. reported comparable findings among Saudi healthcare professionals, where screening practices fell short despite medical training [18].

Barriers to screening were multifactorial, spanning personal, sociocultural, and system-level domains. Notably, lack of guideline awareness was the only barrier significantly associated with non-practice of BSE (p=0.010). Similar barriers, including affordability, unavailability of female physicians, and fear of diagnosis, have been reported across diverse settings [24,25], suggesting that these obstacles are systemic rather than population-specific. Family history emerged as a significant facilitator of BSE practice. Participants with affected relatives were significantly more likely to practice BSE than those without (79.6% vs. 58.3%, p=0.013), suggesting that personal relevance, rather than abstract knowledge, may be the stronger driver of preventive behavior.

These findings carry several implications. First, since knowledge alone did not translate into practice, educational programs must move beyond information delivery to address perceived susceptibility and provide structural support, such as workplace screening programs and reminder systems. Second, the significant association between family history and BSE practice suggests that systematic identification and counseling of women with affected relatives may be an effective, targeted intervention. Third, the finding that lack of guideline awareness was the only barrier significantly associated with non-practice highlights that even physicians require regular updates on current screening recommendations through continuing medical education. Finally, given that both NCCN and ESMO recommend CBE for women aged 25-39, healthcare institutions should facilitate access to clinical breast examination for their own female staff through dedicated wellness or occupational health programs. Qualitative approaches, such as interviews or focus groups, are needed to explore the psychological and contextual factors underlying the knowledge-practice gap. Multi-center studies across Pakistan would help determine the generalizability of these findings.

Strengths of this study include diverse representation across clinical specialties, comprehensive assessment of knowledge across multiple domains, and statistical analysis of knowledge-practice relationships beyond descriptive findings. The focus on female doctors who serve as both healthcare providers and role models addresses an important gap in the literature. Limitations include the single-center design, which may limit generalizability, and reliance on self-reported practices that may be subject to recall or social desirability bias. Additionally, the questionnaire did not distinguish between routine and diagnostic mammography.

## Conclusions

This study demonstrates that female doctors at a tertiary care hospital in Peshawar possess strong awareness of breast cancer warning signs and favorable attitudes toward screening, yet their actual screening practices remain suboptimal. The absence of a significant correlation between knowledge and screening behavior suggests that educational interventions alone are unlikely to bridge this gap. Instead, women with a positive family history perceived themselves to be at higher risk and were therefore more likely to follow recommended breast cancer screening practices.

The low mammography uptake aligns with NCCN and ESMO recommendations, given the young age of participants, but the limited engagement with CBE and inconsistent BSE practice represents missed opportunities for early detection. Barriers such as busy schedules, perceived low risk, and lack of institutional screening programs highlight the need for workplace-based interventions that make screening accessible and routine rather than optional.

These findings call for a shift from knowledge-focused education to structured, institutional screening programs complemented by targeted counseling for at-risk individuals. Female doctors are uniquely positioned as both healthcare providers and role models. Investing in their screening behaviors carries implications that extend well beyond individual health.

## Appendices

Appendix A 

**Table 9:** KAP Questionnaire Credits: Created by the authors: Safina Tanveer, Faiza Gul, Asma Rasool Peerzada, Ayesha Johar, Nida Mumtaz, Naveedul Haq, Safwa Nayab, Muhammad Bilal Elahi, Nawal Qadus, and Ikram Ullah. KAP: Knowledge, Attitudes, and Practices, GP: general practitioner.

No.	Question	Response Options
SECTION I: DEMOGRAPHICS		
1	What is your age (years)?	_____ years
2	What is the highest level of education qualification you have obtained?	MBBS / FCPS-I / FCPS-II / MCPS-I / MCPS-II
3	Please indicate your department of work.	_____________________
4	Are you currently a House Officer or Postgraduate Resident?	House Officer / PG Resident (Year: 1-5) / Consultant
5	What is your marital status?	Single / Married / Separated / Divorced / Widowed
6	Age of marriage?	<25 years / 25–35 years / >35 years
7	Have you or your family had breast cancer?	Yes / No / Prefer not to say
8	Are you registered with a GP for your breast cancer screening?	Yes / No / Prefer not to say
SECTION II: KNOWLEDGE ASSESSMENT		
A. Risk Factors		
1	Does having a history of breast cancer increase the risk of developing it again?	True / False / Don't Know
2	Does using hormone replacement therapy (HRT) increase the risk of breast cancer?	True / False / Don't Know
3	Cigarette smoking increases the risk of breast cancer.	True / False / Don't Know
4	Being overweight (BMI over 25) increases the risk of breast cancer.	True / False / Don't Know
5	Does having dense breast tissue on a mammogram increase the risk of breast cancer?	True / False / Don't Know
6	Having children later in life or not at all increases the risk?	True / False / Don't Know
7	Doing less than 30 minutes of moderate physical activity at least five times a week increases the risk of breast cancer.	True / False / Don't Know
8	Increasing age is a risk factor for breast cancer.	True / False / Don't Know
9	The use of antiperspirants increases the risk of breast cancer.	True / False / Don't Know
10	Disruptions in circadian rhythm (e.g., night shift work or excess light exposure at night) increase the risk of breast cancer.	True / False / Don't Know
B. Signs and Symptoms		
1	A lump or thickening in your breast could be a sign of breast cancer.	True / False / Don't Know
2	Bleeding or discharge from your nipple could be a sign of breast cancer.	True / False / Don't Know
3	Dimpling of the breast skin could be a sign of breast cancer.	True / False / Don't Know
4	The pulling in of your nipple could be a sign of breast cancer.	True / False / Don't Know
5	A change in the shape of your breast or nipple could be a sign of breast cancer.	True / False / Don't Know
C. Screening Methods		
1	How many recognized screening methods are there for the early detection of breast cancer?	1 / 2 / 3 / None
2	Which of the following are recognized screening methods?	BSE / CBE / Mammography / Ultrasound / MRI / None
3	At what age should women begin breast self-examination (BSE)?	15 years / 20 years / 30 years / 40 years
4	How often should a woman perform BSE?	Once a week / Once a month / Every 6 months / Once a year
5	What is the best time to perform BSE?	During menstruation / A week after menstrual period / Any random day / Before going to sleep
6	Which of the following steps are part of performing BSE?	Inspecting in mirror / Feeling breast with hand / Feeling armpit / All of the above
7	At what age should women at average risk begin clinical breast examination (CBE)?	20 years / 30 years / 40 years / Only if symptoms appear
8	How often should CBE be performed for women at average risk?	Every 6 months / Once a year / Every 5 years / Only when symptoms appear
9	At what age should women at average risk begin mammography screening?	20 years / 30 years / 40 years / 50 years
10	How often should mammography be performed for women at average risk?	Every 6 months / Once a year / Every 2 years / Only if symptoms appear
SECTION III: ATTITUDES TOWARD BREAST CANCER PREVENTION		
1	Maintaining a healthy lifestyle is essential for the prevention of breast cancer.	Strongly Agree / Agree / Neutral / Disagree / Strongly Disagree
2	Breast cancer screening should be practiced even by doctors.	Strongly Agree / Agree / Neutral / Disagree / Strongly Disagree
3	Screening should be prioritized despite workload and time constraints.	Strongly Agree / Agree / Neutral / Disagree / Strongly Disagree
4	Breast self-examination (BSE) is a good practice for the early detection of breast cancer.	Strongly Agree / Agree / Neutral / Disagree / Strongly Disagree
5	Clinical breast examination (CBE) is a good practice for the early detection of breast cancer.	Strongly Agree / Agree / Neutral / Disagree / Strongly Disagree
6	Mammography is a useful tool for the early detection of breast cancer.	Strongly Agree / Agree / Neutral / Disagree / Strongly Disagree
SECTION IV: SCREENING PRACTICES		
1	Do you actively monitor your body weight?	Yes / No
2	Do you engage in at least 30 minutes of physical activity, five times per week?	Never / Rarely / Sometimes / Often / Always
3	Do you consider your family history of breast cancer in your personal health planning?	Yes / No
4	If you have children, did you or would you consider breastfeeding to reduce breast cancer risk?	Yes / No / Not applicable
5	Do you practice breast self-examination (BSE)?	Yes / No
6	If yes, how often do you perform BSE?	Once a month / Every 2–3 months / Once or twice a year / Rarely
7	Have you ever undergone a clinical breast examination (CBE) by a healthcare professional?	Yes / No
8	If yes, how often do you undergo CBE?	Yearly / Every 2–3 years / Only when I notice a problem / Rarely
9	Have you ever had a mammogram?	Yes / No / Not applicable
SECTION V: PERCEIVED BARRIERS TO SCREENING		
1	Busy schedule / lack of time	☐
2	Limited exposure to breast cancer patients in your specialty, reducing focus on personal screening	☐
3	Perceived lower risk due to being young or healthy	☐
4	Fear of receiving a serious or life-threatening diagnosis	☐
5	Embarrassment or discomfort with exposure during screening	☐
6	Lack of awareness about BSE, CBE, or mammography guidelines	☐
7	Limited availability of screening facilities beyond government setups	☐
8	Concerns about radiation exposure from mammography	☐
9	Long waiting times for appointments with breast surgeons in hospital setups	☐
10	Lack of structured programs or reminders for doctors to undergo screening	☐
11	Cultural or religious beliefs that make screening difficult	☐
12	Other (please specify): _______________________	☐
